# Biased holoenzyme assembly of protein phosphatase 2A (PP2A): From cancer to small molecules

**DOI:** 10.1016/j.jbc.2022.102656

**Published:** 2022-11-01

**Authors:** Terrance J. Haanen, Caitlin M. O'Connor, Goutham Narla

**Affiliations:** Division of Genetic Medicine, Department of Internal Medicine, The University of Michigan, Ann Arbor, Michigan, USA

**Keywords:** PP2A, cancer, molecular glues, molecular disruptors, biased heterotrimeric formation, reversible phosphorylation, HCC, hepatocellular carcinoma, PTM, posttranslational modification

## Abstract

Protein phosphatase 2A (PP2A) is a family of serine threonine phosphatases responsible for regulating protein phosphorylation, thus opposing the activity of cellular kinases. PP2A is composed of a catalytic subunit (PP2A Cα/β) and scaffolding subunit (PP2A Aα/β) and various substrate-directing B regulatory subunits. PP2A biogenesis is regulated at multiple levels. For example, the sequestration of the free catalytic subunit during the process of biogenesis avoids promiscuous phosphatase activity. Posttranslational modifications of PP2A C direct PP2A heterotrimeric formation. Additionally, PP2A functions as a haploinsufficient tumor suppressor, where attenuated PP2A enzymatic activity creates a permissive environment for oncogenic transformation. Recent work studying PP2A in cancer showed that its role in tumorigenesis is more nuanced, with some holoenzymes being tumor suppressive, while others are required for oncogenic transformation. In cancer biology, PP2A function is modulated through various mechanisms including the displacement of specific B regulatory subunits by DNA tumor viral antigens, by recurrent mutations, and through loss of carboxymethyl-sensitive heterotrimeric complexes. In aggregate, these alterations bias PP2A activity away from its tumor suppressive functions and toward oncogenic ones. From a therapeutic perspective, molecular glues and disruptors present opportunities for both the selective stabilization of tumor-suppressive holoenzymes and disruption of holoenzymes that are pro-oncogenic. Collectively, these approaches represent an attractive cancer therapy for a wide range of tumor types. This review will discuss the mechanisms by which PP2A holoenzyme formation is dysregulated in cancer and the current therapies that are aimed at biasing heterotrimer formation of PP2A for the treatment of cancer.

Reversible protein phosphorylation is a critical posttranslational modification (PTM), regulated by the opposing action of two classes of proteins: kinases and phosphatases. Kinases catalyze the addition of a phosphoryl group onto a target protein while phosphatases catalyze the removal of these same phosphoryl groups. Protein phosphorylation can occur on serine, threonine, and tyrosine residues but the vast majority (98%) occurs on serine and threonine residues (1, 2). The addition or removal of these phosphoryl groups is a central mechanism for the regulation of diverse cellular processes including, but not limited to, the activation state of enzymes, protein subcellular localization, membrane transport, protein–protein interactions, and protein degradation. Importantly, while phosphatases have been viewed simply as the erasers of protein phosphorylation, in reality, they serve as critical regulators of the duration and timing of a phosphorylation signal (2, 3). Proteins often harbor multiple phosphorylation sites, allowing for additional complexity and layering of signal transduction. Within a cell, protein phosphorylation is highly dynamic and regulated in both time and space, allowing for a cell to rapidly respond to a plethora of microenvironmental cues and signals. Overall, balanced and regulated protein phosphorylation is key to cellular homeostasis, and its dysregulation is hallmark to the pathogenesis of many diseases, including cancer.

While there are roughly 500 identified cellular kinases, paradoxically, there have been only approximately 60 identified phosphatases (4, 5). This imbalance led to an early misconception that phosphatases had relatively nonspecific enzymatic activity. Many protein phosphatases, however, are composed of multimeric protein complexes, allowing for the assembly of a repertoire of structurally distinct holoenzymes, explaining the observed difference in the number of kinases *versus* phosphatases present in our proteome. The serine/threonine protein phosphatase 2A (PP2A) is a perfect example of how the structural diversity of protein phosphatases establish both the broad repertoire of target proteins while allowing for substrate specificity for a given heterotrimeric complex. PP2A is serine/threonine-directed phosphatase family, where the active holoenzyme is composed of a scaffolding subunit (PP2A Aα/β), a catalytic subunit (PP2A Cα/β), and one substrate-directing B regulatory subunit. Both the A and C subunits have two isoforms, α and β, with the α isoform being the more highly expressed isoform for both subunits (6, 7, 8). The active site of the catalytic subunit (PP2A C) has two manganese atoms that when bound assists in substrate catalysis by binding to the phosphoryl group allowing for the hydrolysis of serine or threonine phosphate esters (9, 10). The various regulatory B subunits have been divided broadly into four structurally distinct groups: B55/PR55/B, B56/PR61/B′, PR48/PR72/PR130/B′′, and Striatin/PR93/PR110/B′′′. Within this review, we will refer to these subunit families as the first family name listed. Each structural family contains multiple isoforms and splice variants resulting in the ability to form over 60 different PP2A heterotrimers to regulate the activity, subcellular localization, and substrate specificity of PP2A (Fig. 1) (3, 5, 11, 12, 13).

**Figure 1:**
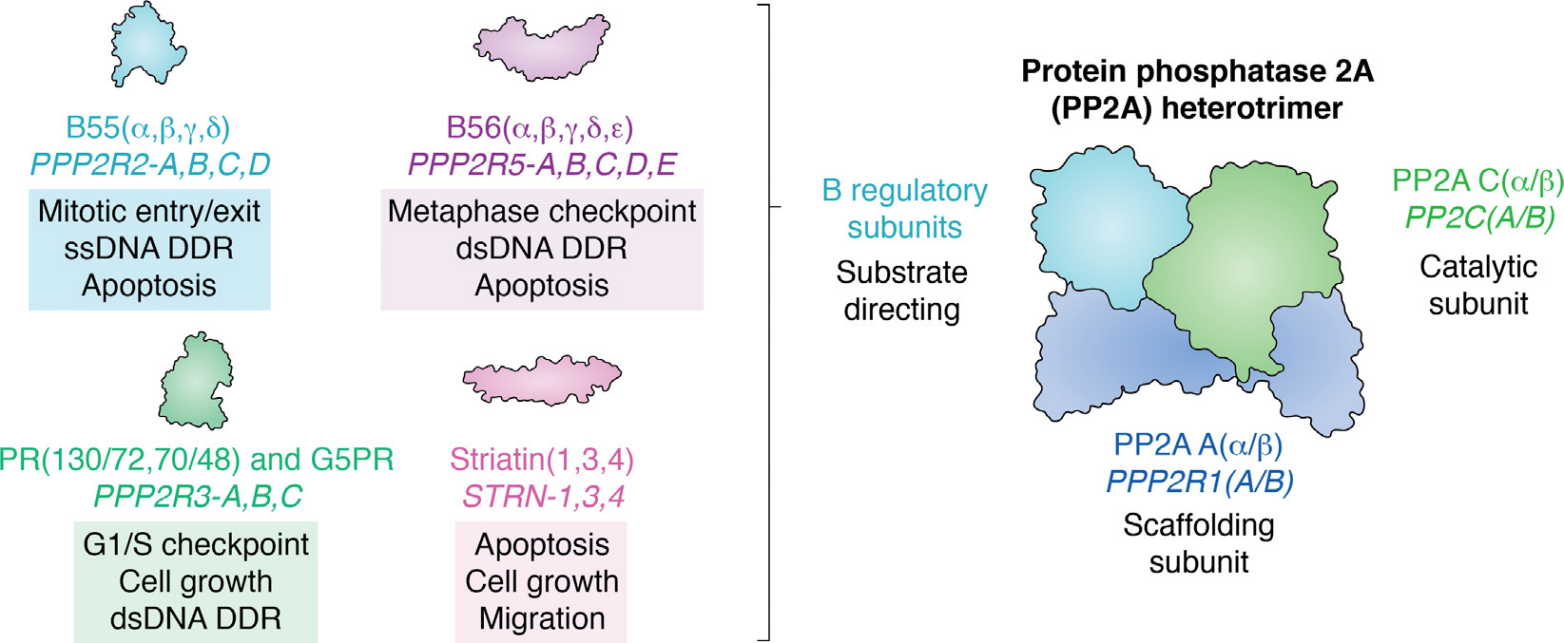
**PP2A is a family of heterotrimeric complexes that regulates many different cellular processes that are crucial for oncogenic transformation.** PP2A is composed of a catalytic subunit (PP2A C) and a scaffolding subunit (PP2A A) with two isoforms (α and β) and various substrate-directing B regulatory subunits. The different families of B regulatory subunits, which can be further subsetted by their respective isoforms (3, 5, 11, 12, 13).

In aggregate, PP2A activity, accounted by all members of the heterotrimeric family, establishes this protein superfamily as a master regulator of a plethora of cellular processes including, but not limited to, cell cycle progression and mitotic exit, the DNA damage response, extracellular mitogenic signals, and cell metabolism (12). Given the critical role PP2A plays in cellular homeostasis, its biogenesis is highly regulated at multiple levels. This regulation starts with unbound PP2A C (13, 14, 15, 16, 17). Free PP2A C binds to alpha 4 (α4) leading to the inactivation of the PP2A C catalytic site and protecting it from degradation thereby preventing any damaging promiscuous unregulated catalytic activity while maintaining an ample reserve of PP2A C that can be reactivated by the PP2A Phosphatase activator (PTPA) to rapidly respond to changes in cellular homeostasis (Fig. 2*A*) (16, 18). PTPA reactivates PP2A C by stabilizing its active site, thus promoting binding of ATP phosphoryl groups, which in turn changes the binding preferences of manganese ions ultimately priming the active site for ATP hydrolysis (18). PP2A activity is also regulated through PTMs. The reversible carboxymethylation of L309 on the C-terminal tail of PP2A C results in a molecular signal, which guides PP2A heterotrimeric formation (19, 20, 21, 22, 23, 24, 25, 26). Protein methylation at this site occurs at the carboxy terminus of the very terminal amino acid of the protein. This is an extremely unique PTM, found in PP2A and closely related phosphatases. The presence of methylation on L309, catalyzed by Leucine Carboxyl Methyltransferase 1 (LCMT-1), blocks the negative charge on the terminal carboxylic acid, allowing for binding of both B55 and B56 family members to the PP2A A/C dimer (Fig. 2*B*). Conversely, Protein Phosphatase Methylesterase 1 (PME-1) mediated removal of this PTM keeps the negatively charged terminal carboxylic acid, resulting in steric hindrance preventing B55 and B56 family member binding. However, some PP2A regulatory subunit families, including the Striatin and PR72/130 families can bind regardless of the carboxymethylation status of PP2A AC heterodimer (Fig. 2*C*).

**Figure 2:**
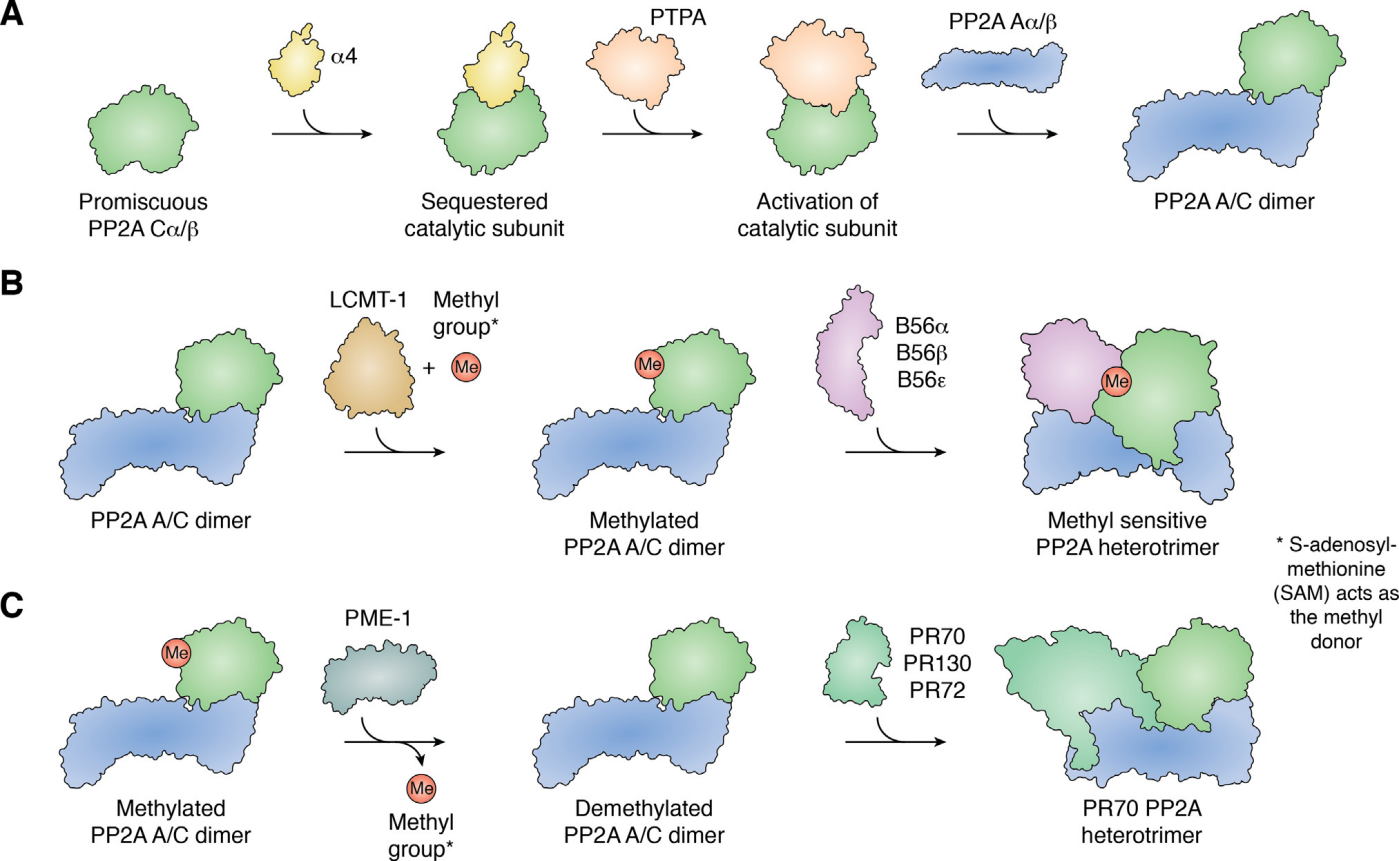
**PP2A biogenesis is highly complex and regulated at multiple levels.***A*, free PP2A Cα/β is sequestered and inactivated by α4 preventing any promiscuous phosphatase activity and generating a reserve of catalytic subunit. In response to cellular stimuli, PTPA will reactivate PP2A Cα/β allowing PP2A biogenesis to begin by binding to PP2A Aα/β to form the PP2A A/C dimer (16, 18). *B*, PP2A biogenesis is further focused through the carboxymethylation of the C-terminal tail of PP2A Cα/β catalyzed by LCMT-1 and ultimately generating a bias in heterotrimeric formation toward B regulatory subunits that show enhanced affinity. The B56 family is shown, but it is not the only carboxymethyl-sensitive B subunit family (41, 47, 55, 71, 74, 81, 82, 83, 84, 85, 86, 87, 88, 89). *C*, conversely, PME-1 acts to remove carboxymethylation shifting the balance of PP2A heterotrimeric formation toward carboxymethylation-insensitive heterotrimers like PR70-PP2A (20, 90).

There have been a number of reviews that have provided comprehensive summaries of PP2A’s role in cancer and how this phosphatase family can be targeted therapeutically (2, 3, 27, 28, 29, 30, 31, 32). Here, we will review and discuss the mechanisms that bias PP2A holoenzyme assembly in human cancer and how recent research has shown that small molecules can be used to stabilize or inhibit specific PP2A holoenzymes as a potential therapeutic strategy for the treatment of a diverse range of cancers.

## PP2A heterotrimer dysregulation in cancer

PP2A was established as a tumor suppressor through the study of okadaic acid and the Simian Virus 40 small T antigen (SV40 ST). These studies demonstrated that loss of PP2A function through either inhibition of its catalytic activity or through the displacement of regulatory B subunits was a key step in driving cellular transformation and tumorigenesis (33, 34, 35, 36, 37). Multiple cellular and mouse models, now supported by patient genetic data, have established PP2A as a haploinsufficient tumor suppressor (38, 39, 40, 41). Interestingly, partial or complete knockdown of only certain PP2A subunits were sufficient to induce oncogenic transformation, suggesting that specific regulatory subunits are responsible for PP2A’s tumor suppressive activities. Furthermore, the Cancer Dependency Map (DepMap.org) identified *PPP2CA* and *PPP2R1A*, which encode the α isoforms of the catalytic and scaffolding subunits of PP2A, respectively, as common essential genes for cellular survival (42, 43). Collectively, this suggests that tumor cells select a window of PP2A activity, maintaining activity essential for cellular function, while selectively inactivating PP2A’s tumor-suppressive functions, allowing for uncontrolled cancer cell growth. The concept of PP2A inactivation in human cancer has been extensively reviewed (40, 44, 45, 46, 47, 48, 49, 50). However, in the sections later, we will focus specifically on how PP2A alterations in cancer lead to biased holoenzyme assembly, allowing tumor cells to capitalize on the progrowth and survival functions of PP2A.

### SV40 small T antigen

As stated previously, one of the key findings supporting PP2A’s role as a tumor suppressor was through the study of the DNA tumor virus SV40. SV40 expresses two key viral antigens, the small and large T antigen. Expression of these viral antigens facilitates human cellular transformation. The large T antigen (SV40 LT) binds and inactivates the tumor suppressor proteins p53 and RB (33, 34, 35, 51). The SV40 small T antigen (SV40 ST) was identified to bind to the PP2A Aα subunit, which remains its only known cellular target (34, 52). Structural data suggested that SV40 ST bound to PP2A Aα and blocked B regulatory subunit binding (34, 35, 45). Depletion of B56γ partially phenocopied SV40 ST expression in the HEK-TER (nontransformed cellular model that expresses SV40 LT, human telomerase catalytic subunit, and oncogenic HRAS allele) model, suggesting that SV40 ST may displace more than one B regulatory subunit (53). Additional studies have shown that depletion of B56α, PR130/PR72, and PTPA in addition to B56γ is sufficient to phenocopy SV40 ST expression in the HEK-TER model, yet depletion of B56β, B56δ, B56ε, PR48, and Striatin 3 is not (45). Supporting the structural data, it was shown that the expression of SV40 ST led to the degradation of B55α, a subunit subject to proteasomal degradation when not bound to A/C (54). Interestingly, SV40 ST was found to associate with PP2A independent of carboxymethylation of the C subunit at L309 (55). Recently, further mechanistic insight into SV40 ST–mediated transformation has been elucidated. The expression of SV40 ST was shown to promote interaction of the PP2A A/C dimer with Striatin 4, a methylation independent regulatory subunit (44). This bias in PP2A heterotrimer formation was shown to facilitate the recruitment of the Striatin Interacting Phosphatase and Kinase (STRIPAK) complex, which redirected PP2A activity to dephosphorylate MAP4K4 and induce cellular transformation through the activation of the Hippo pathway effector, YAP1, a known cellular oncogene (56, 57). Importantly, it was shown that Striatin 4 was required for SV40 ST–mediated cellular transformation (44). Combined, the expression of SV40 ST results in a redirection of PP2A activity, away from the formation of tumor-suppressive holoenzymes and toward a tumor promoting PP2A-STRN4 complex.

### PP2A Aα alterations

Loss of PP2A function through decreased expression or heterozygous somatic mutations of Aα induce tumorigenesis. Complete loss of Aα, however, has been shown to be nonviable in mouse, rat, and *drosophila* models and homozygous mutations in Aα have not been documented in human cancer (42, 58, 59). Furthermore, hemizygous loss of Aα occurs recurrently (42%) in prostate adenocarcinomas, and hemizygous loss of Aα occurs more frequently (greater than 75%) in metastatic disease (40). The loss of Aα scaffold subunit expression has been shown to lead to the decreased expression of B55α in multiple cell lines, and prostate cancer cells with Aα loss were markedly sensitive to B55α reconstitution (40, 60).

While Aα has been shown to have decreased expression in cancer, an additional mechanism by which the Aα subunit is inactivated is through mutation of the scaffolding subunit of PP2A. The scaffolding subunit is a highly flexible protein made up of 15 tandem Hunting Elongation A subunit Tor (HEAT) repeats (48). Based on the crystal structure, it was hypothesized that binding of the various B regulatory subunits and the catalytic subunit was facilitated by highly conserved amino acids located within the intrarepeat loops and that these residues may present a vulnerability that could be exploited by cancer to selectively inactivate the tumor-suppressive functions of PP2A (35). In fact, through both targeted and large-scale genomic sequencing efforts, two mutational hotspots were identified, located within HEAT repeats 5 and 7 of the PP2A scaffolding subunit (46, 49, 50). These mutational hotspots cluster in the region that directly contacts different B regulatory subunits (11, 35, 50, 61, 62). It is important to note that these mutations are heterozygous, leaving the possibility that they might not only result in a loss of PP2A tumor-suppressive function but might also promote gain of function properties.

**Figure 3:**
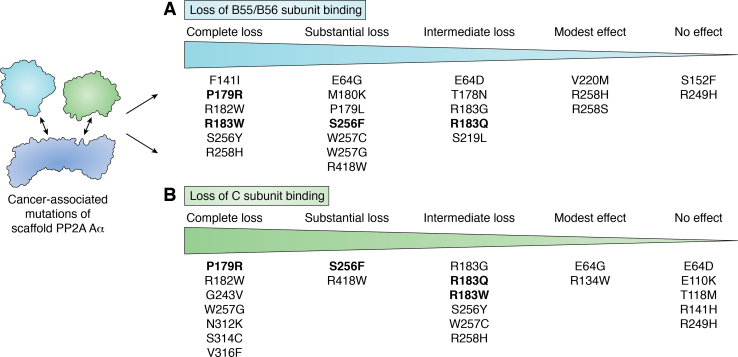
**PP2A Aα mutants effect the PP2A interactome in a graded manner.***A*, the different cancer-associated PP2A Aα mutants can be broken down by the degree to which they lose binding to PP2A Cα/β. *B*, similarly, the mutants can be broken down by the degree to which they lose binding to the B55 and B56 family members. Collectively, this illustrates that the scaffold mutants that demonstrate loss of catalytic subunit often demonstrate coordinate loss of association with the more tumor-suppressive B regulatory subunits (39, 46, 47, 55, 63, 64, 65, 66, 67). Recurrent *PPP2R1A* hotspot mutations with greater than ten independent observations in cancer were bolded to demonstrate the effect on PP2A heterotrimer formation (69).

Further research has been conducted to better understand how and if mutations to Aα alter holoenzyme binding, and a comprehensive summary of all the mutations studied can be found in Figure 3, highlighting that different mutations display varying degrees of impaired subunit binding (39, 46, 47, 55, 63, 64, 65, 66, 67, 68, 69). The mutations studied include those in the mutational hotspots but also those outside of these regions. As illustrated in Figure 3, different mutations to Aα display different degrees of impaired B regulatory subunit binding. Several of the recurrent mutations (P179R, R182W, R183G/Q/W, and S256F) display impaired binding to B regulatory subunits of the B55 and B56 families and display increased binding to Striatin family members (39, 46, 47, 55, 63, 64, 65, 66, 67, 68, 69) (Fig. 3*A*). Interestingly, this pattern of heterotrimer bias mirrors that of SV40 ST, but the dependence of the transformative potential of these Aα mutants on Striatin 4 binding, for example, has not been explored. Additionally, coimmunoprecipitated complexes from both Aα-P179R and Aα-R183W mutant expressing cells displayed reduced PP2A enzymatic activity, even when this activity was normalized for C subunit binding in the complex (63, 64). This suggests that there may be other proteins or mechanisms responsible for their tumor-promoting activity beyond their selective B regulatory subunit loss. In support of this concept, the Aα-S256F mutant demonstrated increased binding to Tip41-like protein (TIPRL), which has been shown to inhibit PP2A activity by binding to the demethylated tail of the C subunit effectively sequestering the catalytic subunit (47, 70). While the hotspot mutations have similar patterns of disruption of B subunit binding, there are important differences for C subunit binding. For example, the Aα-P179R mutation impairs binding to the catalytic subunit disrupting PP2A A/C dimer formation, resulting in catalytic subunit degradation, which is not seen for other mutants (Fig. 3*B*) (47, 64).

The cumulative effect of Aα mutations is a bias in PP2A heterotrimer formation that results in the activation of cellular signaling, which promotes the growth and survival of cancer cells. Studies of the downstream signaling of these mutants suggest that the cellular signaling changes upon mutation of PP2A may occur in both a cell type–dependent and context-dependent manner. For example, in Kirsten Rat Sarcoma Virus (KRAS) mutated cancer cells, it was shown that the Aα-R183W mutation potentiates mitogen-activated protein kinase (MAPK) pathway signaling through increased phosphorylation of Extracellular Signal-regulated Kinase ½ (ERK1/2) (63). However, in endometrial cancer cells, without KRAS mutations, Aα-R183W and other Aα mutants were shown to reduce ERK1/2 phosphorylation and increase AKT phosphorylation (47, 63, 71). Other PP2A scaffold mutants have also been shown to activate AKT signaling as well, including Aα-E64D, resulting in increased lung cancer progression and decreased survival in mice crossed with constitutively active RAS (68, 72, 73, 74). Furthermore, the Aα-R183Q/G, S256F, R182W, and P179R mutants demonstrated enhanced activation of the ribosomal protein S6 kinase, PI3K/AKT/Mammalian Target of Rapamycin (mTOR), and WNT/β-catenin pathways, all of which stimulate protein translation, cellular proliferation and differentiation, and survival (47). Further research investigating the effects of different amino acid changes at one position, for example, R183W *versus* R183Q, will provide key insight into the nuanced differences in downstream signaling driven by specific PP2A scaffold mutations. Additionally, elucidating how the cell signaling in PP2A mutant cells is altered in the presence of different oncogenic drivers may begin to explain some of the tissue specificity of certain PP2A mutations.

### Methylation of the PP2A C subunit

Protein methylation is a PTM that most commonly occurs on arginine and lysine residues; however, in the case of PP2A, methylation of the C-terminal tail of the catalytic subunit occurs at the terminal leucine (L309) residue, a unique reversible methylation event resulting in the formation of a carboxymethyl ester. This modification is regulated by the activity of LCMT-1, the methyltransferase, and PME-1, the methylesterase (10, 74, 75, 76, 77, 78, 79). The presence of carboxymethylation of PP2A C has been reported to have a modest to no effects on PP2A enzymatic activity *in vitro*, initiating inquiries into mechanisms through which this PTM regulates PP2A activity and function (74, 80). Interestingly, it was determined that carboxymethylation of the catalytic subunit is an evolutionarily conserved mechanism to promote the formation of specific heterotrimeric complexes (74, 77, 81). Modification of carboxymethylation, through expression changes in PME-1 or LCMT-1, is another method by which PP2A function is pathologically subverted in cancer (19). Importantly, knockdown of LCMT-1 or overexpression of PME-1 was shown enhance cellular transformation in the HEK TER/B56γ model, resulting in activation of the AKT/S6K pathway (41).

As stated previously, the carboxymethylation status of the catalytic subunit has been shown to alter the binding of specific B regulatory subunits to the PP2A A/C dimer. Specifically, the B55 family, B56α/ε, have been shown to be methylation-sensitive regulatory subunits, indicating that they preferentially bind to the methylated A/C dimer (41, 47, 55, 71, 74, 81, 82, 83, 84, 85, 86, 87, 88, 89) (Table 1). A recent study analyzed the effects of catalytic subunit carboxymethylation of various protein phosphatases on holoenzyme assembly using a proteomics-based approach (82). This study supported the findings that specific members of the B55 and B56 family of regulatory subunits are sensitive to the carboxymethylation status of the catalytic subunit. Furthermore, this study showed within the B56 family, specific regulatory subunits demonstrated differential sensitivity to changes in carboxymethylation of the catalytic subunit. Specifically, B56α and B56ε were more dependent on methylation for binding compared to the B56γ and B56δ regulatory subunits. The authors also found that the PR72 family showed some sensitivity to the C subunit methylation status while Striatin family member binding was unaffected. Interestingly, the Striatin methyl independence was lost in nontransformed cell lines. This change in Striatin carboxymethyl sensitivity requires further investigation, as the authors only used one immortalized nontransformed cell line (82). It is important to note that many of these studies use mutation to the C-terminal tail to decrease PP2A C carboxymethylation but distinguishing the effects of carboxymethylation status from the loss of a direct amino acid interaction with B regulatory subunits remain challenging. A summary of the published studies on the effects of PP2A C mutations on carboxymethylation is presented in Table 2 (41, 47, 55, 71, 74, 81, 82, 83, 84, 85, 86, 87, 88, 89).

**Table 1:** The effect of PP2A Cα mutations on the PP2A interactome

Change to PP2A C	B55 family			B56 family				PR family			Striatin family			Viral antigens			Ref
	α	δ	γ	α	δ	ε	γ	PR130/PR72	PR59	PR70/PR48	Striatin	Striatin 3	Striatin 4	SV40 ST	PyST	PyMT	
H59Q	<0.15										1–0.70	0.40–0.15				1	(55)
D85N	<0.15															1	(55)
	<0.10													1	1		(41)
R89A	<0.15															1	(55)
H118Q	<0.15															1	(55)
T301D	<0.15															1	(55)
	0.5															2	(87)
T301Stop	<0.15										0.70–1	0.70–1				1	(55)
	0															1	(87)
T304A	>2										>2	1–0.7				1	(55)
	1.73 ± 0.45			0.46 ± 0.10	1.13 ± 0.13				0.59 ± 0.09								(90)
	1.79 ± 0.12			0.50 ± 0.11	1.12 ± 0.62				0.60 ± 0.15								(90)
	1.5															0.5	(87)
T304D	<0.15										1–0.70	1–0.70					(55)
	0			0.22 ± 0.6	1.92 ± 0.88				1.51 ± 0.22								(90)
	0			0.22 ± 0.08	2.38 ± 0.94				1.90 ± 0.40								(90)
	0															1	(87)
T304K	<0.10															3	(87)
	<0.15															1	(90)
T304N	<0.15															1	(55)
	0															3	(87)
Y307E	<0.15										>2	>2				1	(55)
	0.02 ± 0.04			0.05 ± 0.07	0.20 ± 0.10				0.69 ± 0.11								(55)
	0			0	0.18 ± 0.05				0.57 ± 0.08								(90)
	0															1	(90)
Y307F	>2										>2	>2				1	(87)
	1.65 ± 0.28			0.05 ± 0.04	0.23 ± 0.11				1.17 ± 0.14								(55)
	1.53 ± 0.07			0.09 ± 0.08	0.42 ± 0.16				1.24 ± 0.22								(90)
	1															2	(90)
Y307K	<0.15															1	(87)
	0															2	(90)
Y307Q	<0.15															1	(55)
	0															1	(87)
	0															1	(90)
ΔL309	<0.15										>2	>2					(55)
	0.04 ± 0.04			0.1 ± 0.1	0.21 ± 0.19				1.52 ± 0.40								(90)
	0			0	0.15 ± 0.11				1.30 ± 0.35								(90)
	<0.20													1	1		(41)
	0	<0.20	<0.10	0	0.8	0.3	0.8	0.6		0.44	0.9	0.9	0.9				(84)
	1																(88)
L309A	0																(76)
	1																(88)
L309Q	0	0	0	0													(91)
mPP2A C	1			1		1											(86)
PME-1 OE	1																(88)
LCMT1 KD	<0.10	0.16		0.42	0.16	0.16	0.66	0.33		<0.05	1.16	1.16	1.16				(84)
	0.22	0.44		0.66	0.44	<0.05	0.55			0.1	1	1	1				(84)
	0	0.25		0.58	0.33	0.5	0.36	0.5		0.08	0.75	0.75	0.83				(84)
ABL127	8000X			0			0	250X									(83)

The table summarizes the results of experiments including but not limited to coimmunoprecipitation, mass spectrometry, liquid chromatography, and *in vitro* reconstruction of PP2A heterotrimers that highlights the effect of point mutations and deletions on PP2A Cα, overexpression of LCTM-1 or PME-1, and the inhibition of PME-1 across the different B regulatory subunit families and viral antigens that comprise the PP2A interactome. Each value indicated represents the fold change in association when compared to the association seen with the WT PP2A Cα (41, 47, 55, 71, 74, 81, 82, 83, 84, 85, 86, 87, 88, 89).

**Table 2:** The effect of PP2A Cα mutations and/or overexpression of LCMT-1 or PME-1 on carboxymethylation

Change to PP2A C	mPP2A C levels	Ref
H59Q	<0.15	(55)
D85N	<0.15	(55)
	<0.10	(41)
R89A	<0.15	(55)
H118Q	<0.15	(55)
T301D	1–0.70	(55)
	0	(87)
T301Stop	<0.15	(55)
	0	(87)
T304A	1–0.70	(55)
	1–0.71	(90)
	1	(87)
T304D	1–0.70	(55)
	1–0.71	(90)
	1	(97)
T304K	1–0.70	(55)
	1	(87)
T304N	1–0.70	(55)
	1	(87)
Y307E	<0.15	(55)
	<0.10	(90)
	0	(87)
Y307F	0.40–0.15	(55)
	0.70–0.41	(90)
	1	(87)
Y307K	<0.15	(55)
	0	(87)
Y307Q	<0.15	(55)
	0	(87)
DL309	<0.15	(55)
	<0.10	(90)
	<0.10	(41)
	0	(84)
	0	(88)
	0.55	(89)
L309A	0	(76)
	0	(88)
L309Q	0	(87)
mPP2A C	1	(86)
PME-1 OE	<0.10	(88)
	0.5	(89)
LCMT1 OE	1.25	(89)
LCMT1 KD	0	(84)
LCMT1 OE + L309Del	0.6	(89)
LCMT1 OE + PME1 OE	0.25	(89)

The table summarizes the results of experiments including but not limited to coimmunoprecipitation, mass spectrometry and *in vitro* reconstruction of PP2A heterotrimers that highlights the effect of PP2A Cα point mutations and deletions on the levels of carboxymethylation. Each value posted represents the fold change in levels of carboxymethylation when compared to the carboxymethylation levels observed by the WT PP2A Cα (41, 47, 55, 71, 74, 81, 82, 83, 84, 85, 86, 87, 88, 89).

In the context of cancer, LCMT-1 and PME-1 have been shown to act as regulators of oncogenic signaling through the modulation of PP2A carboxymethylation. Importantly, knockdown of LCMT-1 or overexpression of PME-1 was shown enhance cellular transformation in the HEK TER/B56γ model. Specifically, it was shown that LCMT-1 knockdown resulted in decreased PP2A C subunit carboxymethylation and activation of the AKT/S6K pathway and that this activation of AKT signaling was necessary for the enhanced cellular transformation observed in this model system (41). Conversely, overexpression of PME-1 resulted in decreased carboxymethylation and increased AKT phosphorylation (20, 90). In another study, PME-1 overexpression in glioma cells resulted in increased MEK1/2 phosphorylation and activation of downstream signaling proteins through the increased association of PP2A with the MEK1/2 complex (90). Similar to the studies on PP2A scaffolding point mutations, these changes in cellular signaling may be context dependent, as the overexpression of PME-1 promoted both ERK and AKT signaling in endometrial cancer and glioma cells, but this was not observed in colorectal cancer cells (20, 91). Combined, the effects of LCMT-1 and PME-1 expression on cellular transformation support the central role for PP2A in regulating tumorigenesis and phenocopies results obtained with individual regulatory subunit knockdown in these same models of cellular transformation.

The levels of carboxymethylated PP2A has been correlated with cancer progression and both progression free and overall survival in patient cohorts. Decreased PP2A carboxymethylation has been associated with increased cancer progression possibly as a result of loss of specific B subunit binding to the carboxymethylated PP2A A/C dimer (21, 92). Additionally, modulation of PP2A carboxymethylation has been studied in the context of cancer therapeutic response, where overexpression of PME-1 was found to drive kinase inhibitor resistance in glioma cells and knockdown of PME-1 increased sensitivity to epidermal growth factor receptor (EGFR) and Polo-Like Kinase 1 (PLK1) inhibitors in lung adenocarcinoma cells (93).

Cancer cells increase the metabolism of glucose to produce increasing amounts of lactate to generate sufficient ATP to fuel the tumorigenic cellular pathways that drive cancer growth (94).The increased consumption of glucose in cancer cells may modulate PP2A activity *via* regulation of carboxymethylation of the catalytic subunit (61, 93, 95). Interestingly, the scaffolding subunit that acts to immobilize and stabilize the catalytic subunit to promote LCMT-1 interaction and drive enhanced carboxymethylation of the catalytic subunit is the most frequently mutated subunit of PP2A in cancer, suggesting that cancer selects multiple mechanisms to disrupt PP2A carboxymethylation (61).

It is important to note that there are limitations in the tools available for detecting the carboxymethylation status of the C-terminal tail of the catalytic subunit, as there have been studies that have shown commonly used antibodies are unable to discriminate between the C terminus of PP2Ac, PP4c, and PP6c as a result of the high degree of sequence homology between these phosphatases. Additionally, some of the commonly used antibodies are not specific to the carboxymethylated form of the catalytic subunit (80, 96, 97, 98, 99, 100). Careful assessment of antibody specificity and the inclusion of experiments to distinguish between the protein phosphatase family members is critical when analyzing carboxymethylation using these antibody-based approaches.

### Altered expression of PP2A B regulatory subunits

PP2A’s enzymatic activity is directed through its association with the different families of B regulatory subunits, making them critical targets for cancer progression. In cancer, global PP2A function is modulated through the downregulation of tumor-suppressive regulatory subunits while other growth promoting subunit classes are maintained.

#### B55 family members

The B55 family of regulatory subunits consists of *PPP2R2A* (B55α), *PPP2R2B* (B55β), *PPP2R2C* (B55γ), and *PPP2R2D* (B55δ). B55α is one of the most abundantly expressed B regulatory subunit, and its expression is closely tied to its ability to bind to the PP2A A/C dimer (60, 101, 102, 103). Threonine 308 of AKT is a reported phosphorylation site regulated by PP2A-B55α, and loss of B55α has been shown to increased T308 phosphorylation and sensitivity to AKT inhibitor treatment (104). Conversely, B55α has been found to be overexpressed in pancreatic ductal adenocarcinoma tissues, and increased expression correlates with worse patient outcome, and depletion of B55α in pancreatic cancer cells lead to a reduction in mitogenic signaling and decreased tumorigenicity, following orthotopic implantation (100). B55β has also been found to be downregulated in 90% of colon cancer cell lines through the hypermethylation of its promoter (105). Low B55γ expression has been documented to promote prostate cancer growth independent of androgen receptor–mediated transcriptional programs, is found in primary and metastatic prostate cancer tumors, and is associated with poor disease outcome, suggesting that it plays a critical role in prostate cancer progression (106). B55δ is downregulated in hepatocellular carcinoma (HCC) tumors and cell lines through the upregulation of miR133b (107). Cisplatin treatment for advanced HCC patients led to the upregulation of B55δ, which resulted in G1 cell cycle arrest, inhibition of cell migration, and the induction of apoptosis. These phenotypes were subsequently lost by depleting B55δ, while overexpression of this regulatory subunit decreased tumor growth *in vivo* (107).

#### B56 family members

The B56 family of regulatory subunits consists of *PPP2R5A* (B56α), *PPP2R5B* (B56β), *PPP2R5C* (B56γ), *PPP2R5D* (B56δ), and *PPP2R5E* (B56ε). It was found that depletion of B56α and B56γ in the HEK TER V model induced cellular transformation and tumorigenesis to a similar extent seen in the HEK TER ST model (45, 53, 108, 109). Elevated expression of B56α correlated with better prognosis in HCC patients. Heterozygous loss, frameshift deletions, and alternative splice isoforms have been found to be enriched in endometrial carcinomas, colon adenocarcinomas, and prostate cancer (110, 111, 112). Interestingly, hotspot mutations in the RNA splicing factor, SF3B1, produce aberrant *PP2R5A* transcript, resulting loss of B56α expression and increased stability of oncoproteins c-*myc* and BCL2 (113). Activation of PP2A *via* FTY-720 (inhibitor of the PP2A inhibitor protein SET) treatment and the overexpression of B56α was sufficient to impair mutant SF3B1 tumor progression (113, 114, 115). Increased expression of the miRNA’s that repress *PPP2R5A* (B56α) transcription, miR-338-5P and miR-218, were found to deplete expression of B56α expression in cancer (116, 117). Recently, investigators sought to establish an independent prognostic marker profile to better identify high risk HCC patient populations (118). This work led to establishment of a five gene profile, including mutations in *PPP2R5B* (B56β) that induced endoplasmic reticulum stress and demonstrated the ability to serve as a prognostic biomarker to identify patients that may present with poorer prognosis (118). B56γ expression was found to be downregulated in lung cancer, and concordantly, overexpression of B56γ has been shown to be tumor suppressive in multiple cancer models (lung, breast, and prostate cancer cell lines) (108, 119). B56δ is not commonly found to be altered in human cancers, but its function appears to be critical for regulating tumorigenesis, as depletion of B56δ results in spontaneous tumor formation in mice (120). These mice develop hematologic malignancies early in life while older mice developed HCC at high frequencies (120). Lastly, aberrant expression of miR-23a leads to the downregulation B56ε resulting in the suppression of apoptosis, thus promoting gastric cancer cell growth (121).

#### PR family members

The PR family consists of *PPP2R3A* (PR130/PR72), *PPP2R3B* (PR70/48), and *PPP2R3C* (G5PR). The *PPP2R3A* gene is unique and contains two start sites, which encode for the PR130 and PR72 subunits (122, 123, 124). PR130 directs PP2A activity toward tumor promoting and suppressive means in a context-dependent manner across cancer, as elevated expression of PR130 may drive cancer progression by accelerating proliferation and enhancing migratory behavior in HCC while depletion of PR130 in fibrosarcoma, breast cancer, and colorectal cancer cells augmented cellular adhesion to collagen I, resulting in impaired cell migration (125, 126, 127, 128, 129, 130). Additionally, PR130-PP2A may also act as a tumor suppressor, as it is found to be epigenetically silenced in acute lymphoblastic leukemia and depletion of PR130 was sufficient to phenocopy the expression of SV40 ST in human cell transformation (45, 126, 131). Depletion of PR130 by miR-652 and its epigenetic silencing mediated by histone deacetylases (HDAC’s) -1, 2, and 3 promote cancer cell growth and migration while leading to the accumulation of DNA replicative stress due to loss of PR130-PP2A regulation on ATM, ultimately resulting in sustained suppression of replicative stress-induced checkpoint signaling cascade (129, 132, 133, 134, 135). Furthermore, the combination of DNA-damaging agents and HDAC inhibitors lead to synergistic cell death and the accumulation of *PPP2R3A* transcripts, suggesting that the depletion of PR130 expression or aberrant expression of HDAC’s may provide an prognostic marker for this combination treatment (131). *PPP2R3B* (PR70) is located on the X and Y chromosomes in the homologous region known as the pseudoautosomal region (PAR) that escapes X chromosome inactivation. Mutations in *PPP2R3B* in human cancer are rare; however, it has been documented that loss of the inactive X chromosome is high in females with breast, ovarian, and melanoma tumors (109). Loss of PR70 correlates with shorter overall survival in melanoma patients, and its depletion enhances tumorigenicity in metastatic melanoma models (109). Depletion of PR70 may induce the onset of mitotic catastrophe, given its role in maintaining genomic stability through its negative regulation of the retinoblastoma (Rb), thus preventing inhibition on DNA synthesis and cell cycle progression (136). Inhibiting PR70 in cancer cells with evidence of mitotic catastrophe or excessive chromosomal instability may be an attractive therapeutic approach as the depletion of PR70 induced cell death in breast, pancreatic, ovarian, glioblastoma, and prostate cancer cells harboring increased polo-like kinase 1 (PLK1) expression (136, 137). To date, there have been no reports of altered G5PR (*PPP2R3C*) expression in cancer; however, its role in the maturation of B-cells has been well investigated and may represent an attractive candidate for holoenzyme stabilization to drive an robust immune infiltration response in vulnerable cancer subtypes (138, 139, 140, 141).

#### Striatin family members

The Striatin family consists of STRN (Striatin), STRN3 (Striatin 3), and STRN4 (Striatin 4). Striatin has been found to be overexpressed in HCC tissues when compared to adjacent nontumor tissues (142). Interestingly, the increase in Striatin expression has been shown to correlate to the cellular reprogramming event, the epithelial to mesenchymal transition, which has been implicated in promoting tumor initiation and metastasis in a variety of epithelial-based cancers (142, 143, 144). It was found that increased Striatin expression correlated positively with the mesenchymal marker vimentin and negatively with the epithelial marker E-cadherin (144). Striatin 3 upregulation resulted in hyperactivation of YAP1, leading to loss of Hippo tumor-suppressive functions, which ultimately correlated with poor prognosis in a cohort gastric cancer patients (143). Lastly, Striatin 4 has been documented to be highly expressed in a variety of cancers, and studies have shown that depleting its expression leads to a reduction in proliferation, invasion, and metastasis both *in vitro* and *in vivo* (144, 145). As mentioned previously, Striatin 4 was recruited by SV40 ST to the Striatin Interacting Phosphatase and Kinase (STRIPAK) complex that redirects PP2A function toward the dephosphorylation of MAP4K4, leading to the activation of YAP1 and the inactivation of Hippo tumor-suppressive function (44).

### ARPP16/19

c-AMP Regulated Protein 16/19 (ARPP16/19) are intrinsically disordered proteins that have been documented to inhibit PP2A function. These proteins do not form stable tertiary structures but instead form a diverse array of transient secondary structures, which are dictated by their binding partner. ARPP16 binds to the Aα subunit (HEATS 2–8), the same region where B regulatory subunits bind, and may influence holoenzyme formation or act as a chaperone to redirect PP2A activity (146, 147, 148).

ARPP-16/19 is a critical cell cycle regulator, and its association with Aα and the B56 family of regulatory subunits is regulated through phosphorylation (148, 149). MAST3 kinase phosphorylates ARPP-16 at S46 and ARPP-19 at S62 to inhibit PP2A function during cell cycle progression to allow for mitotic entry (at the DNA damage checkpoint recovery). Phosphomimetic mutations in ARPP-16 S46E and S62E were shown to enhance affinity toward B56δ and B56α, respectively. ARPP-16/19 has been found to be overexpressed in a variety of aggressive cancers such as HCC and acute myeloid leukemia (146, 148, 149, 150). Thus, ARPP-19 overexpression in these cancers results in inhibited PP2A activity promoting accelerated passage through the cell cycle (146, 150, 151, 152, 153).

In summary, PP2A biogenesis is highly regulated to ensure that its enzymatic activity is not promiscuous, which would disturb cellular homeostasis. In cancer, we observe that this biogenesis is hijacked through a variety of methods, which points to PP2A’s central role as a tumor suppressor. The modulation of PP2A biogenesis occurs through both genetic and nongenetic mechanisms, including the upregulation of negative modulators of PP2A biogenesis and through somatic mutations in the scaffolding subunit that disrupts PP2A A/C dimer formation and B regulatory subunit binding. The cumulative effect of these alterations is the promotion of biased PP2A heterotrimeric formation that promotes oncogenic signaling.

## Therapeutic targeting of PP2A

Cellular homeostasis is dysregulated in human cancer as a result of an imbalance in protein phosphorylation, resulting in global perturbation of cellular signaling pathways. Constitutive kinase activity contributes to this imbalance, and kinase inhibition has become a mainstay of anticancer therapy. The therapeutic targeting of phosphatases, like PP2A, to reestablish cellular homeostasis has been limited by the structural diversity of this family of enzymes. Complicating this approach further, as explained previously, is that certain PP2A holoenzymes are important for its tumor-suppressive activity, while other holoenzymes promote oncogenic transformation (such as STRN4) (44, 63, 64, 80, 83, 142, 143, 144, 145). Thus, there is a need for a more comprehensive understanding of the role of each holoenzyme complex in cancer, as well as high resolution structures of each heterotrimer to allow for specific therapeutic targeting of disease relevant PP2A heterotrimers. Currently, there have only been 5 of the over 60 PP2A holoenzymes (not including splice variants) solved using both X-ray crystallography, and more recently, cryo-EM (9, 11, 12, 143, 154, 155, 156, 157). While the available structures are limited, it is without question that the available structural data on PP2A have aided in the advances toward targeting PP2A therapeutically and will remain a crucial piece for understanding PP2A’s diverse functions.

Targeting protein–protein interactions that facilitate PP2A heterotrimeric formation may present opportunities for the development of small molecules or synthetic peptides that can act as either molecular glues or disruptors. A classic example of a small molecule that acts as a molecular glue for the treatment of cancer is Rapamycin, which binds to mTOR immunophilin, FK-binding protein 12 (FKBP12), stabilizing the FKBP12-mTOR interaction, and subsequently, inhibiting mTOR activation. Rapamycin has been approved by the FDA for mTOR driven cancers (158, 159). Conversely, molecular disruptors have demonstrated clinical efficacy in reactivating tumor suppressors and inactivating oncogenes through targeted disruption of protein–protein interactions. The highly selective molecular disruptor, Venetoclax, prevents the binding of prosurvival proteins like BAX or BAK from binding to BCL2, thereby reactivating apoptotic signaling pathways in BCL2 overexpressing cancers. Venetoclax has been FDA approved for the treatment of lymphocytic leukemia (CLL) and acute myeloid leukemia (160). MYCMI-6 inactivates the oncogene, c-*myc*, by binding to the MAX binding domain (bHLHXZip domain), preventing the formation of the MYC–MAX complex and inhibiting c-*myc* transcriptional activity (161). Collectively, these small molecules highlight the potential for molecular disruptors as an anticancer strategy. To this end, molecular glues and disruptors represent a new avenue for protein phosphatase targeting. In the following section, we will highlight recent studies demonstrating modulation of the PP2A heterotrimer formation using both methodologies. Ultimately, the goal of these approaches is to drive an antitumor response through the stabilization or destabilization of specific PP2A heterotrimeric complexes, resulting in the creation of a new class of anticancer drugs.

### Molecular glues

PP2A’s structural diversity uniquely lends itself to targeted modulation of its phosphatase activity through stabilization of specific heterotrimeric complexes using small molecules. Broad chemical and genetic screens found that a class of antipsychotic drugs, phenothiazines, possessed anticancer properties by inhibiting PKC and the PI3K/AKT signaling pathway, resulting in relocalization of the transcription factor FOXO1 (162, 163, 164). Further investigation into the phenothiazine mechanism of action found that treatment of T-cell acute lymphoblastic leukemia models resulted in PP2A-dependent degradation of MYC, a well-documented PP2A substrate (165). Reengineering of the phenothiazines to separate out the antipsychotic GPCR-related pharmacology from the anticancer PP2A-activating component of this small molecule series was successful and resulted in the generation of a new class of small molecules (165, 166). Further optimization of these small molecules led to the development of small molecule activators of PP2A (SMAPs), which retained the ability to dephosphorylate PP2A substrates and impair tumor growth *in vivo* (167, 168, 169, 170, 171). Additional studies found that SMAPs impaired tumor growth in multiple different cancer types including Burkitt’s lymphoma, pancreatic ductal adenocarcinoma, lung, and breast cancer that are driven by different oncogenes like KRAS and MYC, suggesting that PP2A reactivation may be effective for the treatment of a broad range of cancers (168, 169, 170, 171, 172).

SMAPs function to reactivate specific PP2A heterotrimers by interacting with a discrete binding pocket that is created through the interaction of PP2A Aα scaffolding subunit, the PP2A catalytic subunit, and the regulatory B56α subunit. Disruption of this binding pocket confers SMAP resistance, suggesting that these molecules may induce cancer cell death and impede tumor growth by acting like a molecular glue to promote the formation of discrete heterotrimeric complexes (168, 169, 170, 171). A lead SMAP compound, DT-061, maintained the same principal mechanism of action with optimized pharmaceutic properties to augment *in vivo* potency (166, 168, 171). Interestingly, DT-061 penetrates the blood–brain barrier allowing it to be used for the treatment of metastatic cancers to the brain and for primary brain tumors (171).

**Figure 4:**
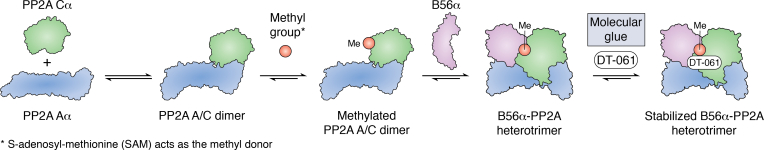
**Targeting PP2A dysregulation using molecular glues.** DT-061 is a PP2A molecular glue that binds to PP2A Aα and promotes the carboxymethylation of PP2A Cα. The formation of the B55α or B56α-PP2A heterotrimer is then selectively stabilized by DT-061 binding (154).

Further mechanistic studies highlighted that DT-061 selectively binds to a unique pocket at the interface between the PP2A Aα, Cα, and B56α subunits through cryo-EM. DT-061 binding stabilized the B56α PP2A holoenzyme and increased PP2A C subunit carboxymethylation (Fig. 4) (154). *In vivo*, DT-061 treatment showed an inverse relationship between the expression of the oncogene c-*myc* and B56α heterotrimer carboxymethylation. The specificity of DT-061 to a single member of the B56 family illustrates that small molecules can be used to selectively stabilize specific PP2A holoenzymes and highlights that PP2A activity can be redirected toward more tumor suppressive substrates.

While DT-061 can be viewed as an exemplar of a reversible PP2A modulator, there are also covalent modifiers being studied. Grossman *et al.* identified a covalently acting small molecule, withaferin A, that binds to cysteine 377 of PP2A Aα at the interface between the PP2A A, C, and B56γ subunits (156, 173). The affinity and potency of withaferin A was optimized to generate JNS-140. While the authors demonstrated that JNS-140 activated PP2A and reduced tumor growth with no signs of toxicity *in vivo* (Fig. 4*C*), they did not define whether increased B56γ-PP2A formation was responsible for the observed increase in PP2A activity or validate that JNS-140 treatment stabilized the formation of this holoenzyme. A remaining question is whether specific holoenzymes can be stabilized by covalently modifying different cysteine residues that may be present within the discrete binding pockets of the different heterotrimers. If covalently modifying molecular glues can stabilize select tumor-suppressive heterotrimers, then they may prove to be a powerful anticancer therapy, since the targeted heterotrimer will be potentially biased for as long as the modified scaffolding subunit is not degraded. However, biasing heterotrimeric formation toward a specific holoenzyme for such an extended period may induce further imbalance in cellular homeostasis, a topic that has yet to be fully explored.

Through extensive experimentation, the PP2A field has been able to map the different protein–protein interaction points that stabilize many different PP2A holoenzyme, thereby leading to the development of small molecules that can fit within these pockets to act as glues. Both DT-061 and iHAP1 represent clear exemplars of molecular glues that can redirect PP2A activity through the selective stabilization of tumor-suppressive holoenzymes. This is an important step forward for the field, as it highlights the ability to develop selective small molecule glues to modulate PP2A heterotrimerization to restore cellular homeostasis through inhibition of pathological phosphorylation.

### Molecular disruptors

**Figure 5:**
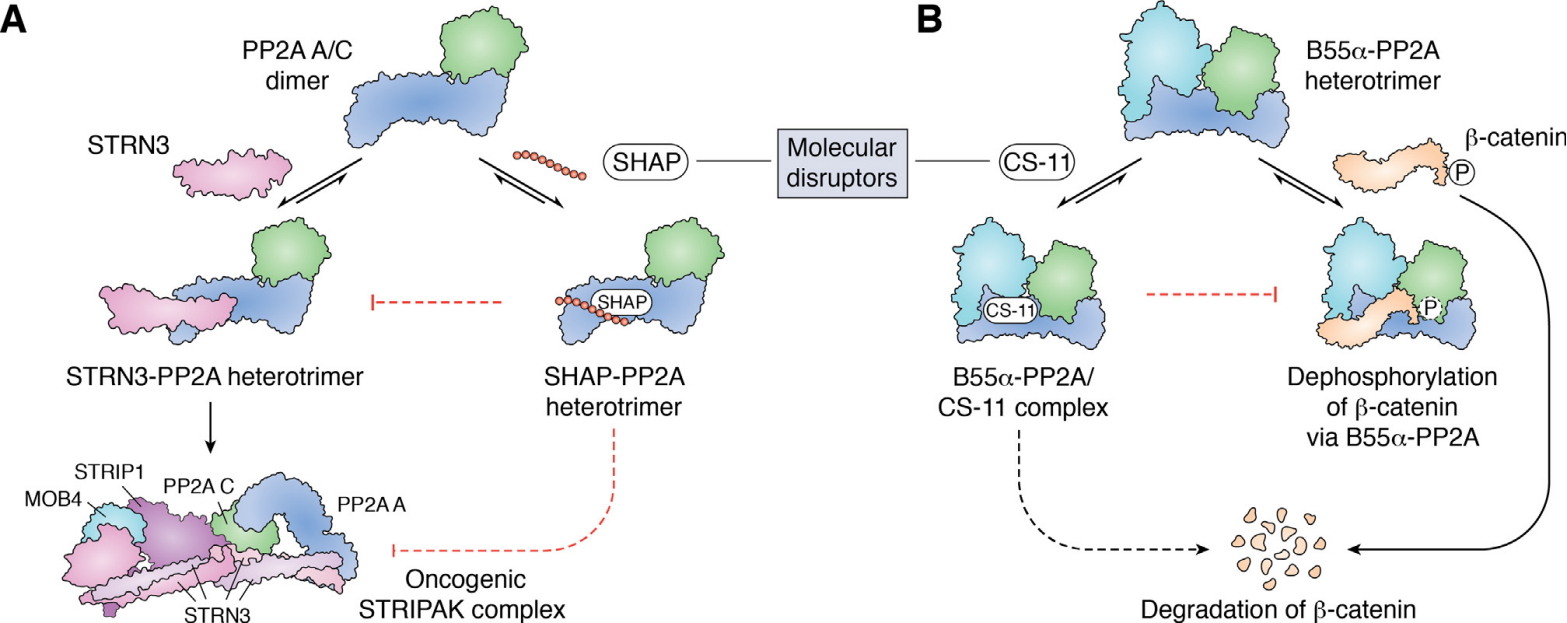
**Targeting PP2A dysregulation using molecular disruptors.***A*, the synthetic peptide, SHAP, competes with STRN3 for binding to the PP2A A/C dimer, thus preventing the formation of the STRN3-PP2A holoenzyme. By extension, SHAP could theoretically prevent the formation and function of the oncogenic STRIPAK complex by displacing STRN3 from the heterodimer, and even if assembled, the SHAP peptide could render the PP2A complex inert (143). *B*, CS-11 is a PP2A molecular disruptor aimed at dissociating β-catenin from the B55α-PP2A heterotrimer, ultimately promoting the degradation of β-catenin by preventing its dephosphorylation (182).

The Striatin family of B regulatory subunits remains one of the most poorly understood PP2A substrate driving subunits in cancer. As highlighted previously, while PP2A is primarily thought to be tumor suppressive, certain holoenzymes, including the Striatins, may be growth promoting and oncogenic (174, 175). The Striatin family members (Striatin, Striatin 3, and Striatin 4) induce the formation of a noncanonical PP2A STRIPAK complex (175, 176). STRIPAK complexes have been implicated in driving tumorigenesis in gastric and colon cancer through negative regulation of the tumor-suppressive Hippo pathway (157, 177, 178, 179). In support of these observations, Striatin 3 has been found to be overexpressed in human gastric cancers, leading to inactivation of the Hippo pathway and hyperactivation of the proto-oncogene YAP1. Based on these findings, Tang *et al.* generated a synthetic peptide named STRN3-derived Hippo-Activating Peptide (SHAP) that competed with endogenous PP2A-Striatin 3 holoenzyme formation (Fig. 5*A*). The authors sought to prevent the PP2A-Striatin 3 heterotrimer from deactivating the tumor-suppressive Hippo pathway (143, 175, 180, 181). PP2A-Striatin 3 dephosphorylates MST1/2, which results in increased YAP1 activation and upregulation of YAP target genes. Synthetic peptides are prone to rapid degradation and have difficulty entering cells; however, SHAP demonstrated high solubility, robust stability, and was efficient in entering cells (143). SHAP are still under development and represent an intriguing method to restrict oncogenic PP2A activity. Specifically, this approach, the inhibition of specific holoenzyme formation could be leveraged toward re-establishing balance in PP2A activity in patients that harbor the Aα-P179R or other PP2A Aα mutations that demonstrate marked loss of binding of tumor-suppressive B regulatory subunits (B56α and B55α) but had no effect on the association with Striatin family members.

Alternatively, molecular disruptors have been used to prevent specific substrates from binding PP2A holoenzymes. The molecule CS-11 was shown to potently inhibit WNT signaling by competing with β-catenin for binding to B55α (182). CS-11 may inactivate the c-*myc* signaling pathway by blocking the PP2A-B55α holoenzyme from dephosphorylating the β-catenin phosphosites that promote its degradation (Fig. 5*B*). In mouse models, CS-11 treatment impaired primary tumor growth and metastases in colorectal adenocarcinoma xenograft models with no overt signs of toxicity. However, given the critical role of WNT signaling and β-catenin in normal physiology, careful toxicology studies will need to be performed. This therapeutic strategy could be used to target c-*myc* driven cancers by disrupting the B55α-PP2A/β-catenin axis. A potential benefit of CS-11 is that it allows for the formation of all PP2A holoenzymes but selectively inhibits the active phosphatase from interacting with specific substrates. Selectively blocking the PP2A-mediated dephosphorylation of tumorigenic substrates may demonstrate the greatest efficacy in cancers that have been documented to be driven by these specific oncogenic substrates.

PP2A has been targeted using small molecules, which impact holoenzyme formation that do not fall into the definition of molecular glues/disruptors. As discussed previously, the overexpression of PME-1 redistributes the balance of PP2A biogenesis toward carboxymethylation insensitive holoenzymes. Inhibitors of PME-1 methylesterase activity have been developed, including aza-β-lactams (ABL)–127, a covalent inhibitor of the PME-1 catalytic site (27, 183, 184). ABL-127 treatment results in increased levels of carboxymethylated PP2A Cα/β, resulting in inhibition of malignant phenotypes such as invasiveness in endometrial cancer cell models. While ABL-127 does not fit into the category of molecular glues or disruptors, its effect on carboxymethylation levels of PP2A Cα/β has an impact on heterotrimer assembly. Specifically, the B55α and PR72 heterotrimer formation was prompted following treatment (Table 1) (83). The entirety of ABL-127’s impact on the PP2A interactome remains incompletely investigated and the impact on additional regulatory subunit binding would be of interest to the cancer field, given the importance of PP2A Cα/β carboxymethylation of the formation of potentially tumor-suppressive PP2A holoenzymes (27, 83, 184). Small molecules that modulate the function of carboxymethylation regulators, like ABL-127, may be an additional class of compounds which can alter the composition of PP2A heterotrimer assembly for cancer treatment.

While not targeting PP2A directly, studies have been performed to determine the effects of PP2A activity on sensitization or resistance to different therapeutics. Specifically, it was shown that inhibition of PP2A drives resistance to the MEK1/2 inhibitor AZD-6244 (63, 172, 185). Additionally, loss of PTPA was found to sensitize to mTOR inhibitors, loss of B55α (*PPP2R2A*) and B56α (*PPP2R5A*) sensitized to poly adenosine diphosphate-ribose polymerase inhibitors (PARPi) (veliparib), Checkpoint Kinase 1 (Chk1) inhibitors (SB218078), and ATR inhibitors (185, 186, 187, 188, 189, 190). Lastly, PP2A Aα mutants were found to sensitize to ribonucleotide reductase inhibitors, including Gemcitabine and Clofarabine (191). Collectively, these approaches illustrate that PP2A-inactivated tumors may be targeted using synthetic lethal based strategies, and PP2A activity may play a significant role in the development of therapy resistance.

Protein phosphatases can coordinately regulate many different key signaling pathways and cellular processes through the structural diversity that characterizes this protein family. PP2A is a perfect exemplar of this concept, as there are over 60 possible PP2A heterotrimers derived from the different B regulatory subunits that drive substrate specificity. Ultimately, developing PP2A-targeting therapeutics and exploring their mechanisms of action will lead to a better understanding of PP2A biogenesis and structure, allowing for the development of new drug classes. This is especially critical in cancer biology, as PP2A activity is found to be inactivated through both genetic and nongenetic mechanisms.

### Future directions for therapeutic targeting of PP2A

Targeting complex formation is a new approach for PP2A and protein phosphatases in general. While the idea of holoenzyme-specific disruptors or glues is a provocative approach, there may be some caveats. First, many of the B subunit isoforms share high sequence homology, indicating that there may be some redundancy in their function. There is some data to support this, as knockdown of certain regulatory subunits in cells results in a reciprocal upregulation of other isoforms from the same family (60, 104, 105, 106). This may suggest that these molecular glues or disruptors may create enough selective pressure to upregulate other isoforms within the same family, which has not been fully explored. However, this redundancy may not be the same in an *in vivo* setting. For example, homozygous KO of Aα is embryonic lethal in mice but can be achieved in cells by CRISPR due to an upregulation of Aβ, indicating that in a mouse, upregulation of Aβ cannot fully compensate for its loss (43, 60). Similarly, complete KO of B55α is lethal, while KO of the β, γ, and δ isoforms are all viable (59). More studies will need to be completed in order to understand the complexity of the functional redundancy of each subunit, as conceptually this has the potential to be a limiting factor for the efficacy of these molecules. A mentioned previously, structural data is not available for all PP2A holoenzymes. Given the similarity between some of the regulatory subunit isoforms, it is possible that there may be both unique and shared interfaces that exist. With a more complete series of high-resolution structures, it may be possible to target shared interfaces (to potentially overcome isoform redundancy) or unique interfaces when desired. Regardless of these caveats, as with all drug candidates, the success of these compounds will ultimately need to be determined in the clinic.

## Conclusion

**Figure 6:**
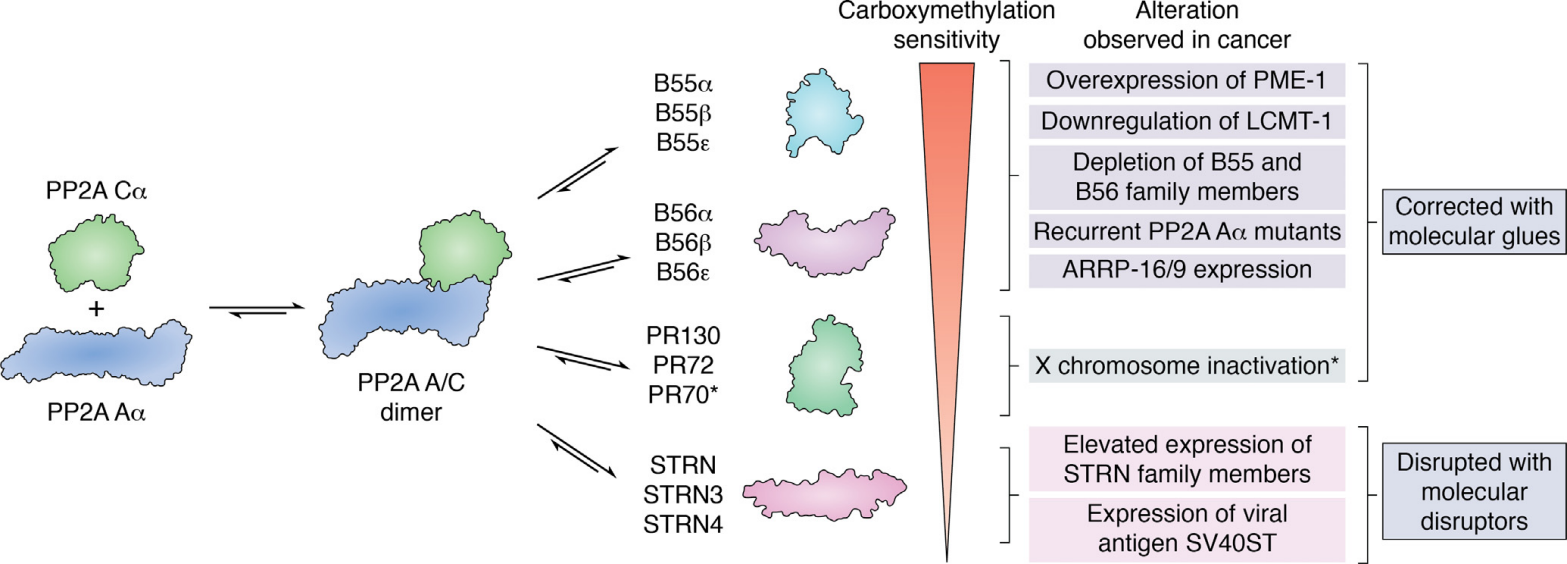
**PP2A is a family of serine/threonine phosphatases composed of the PP2A Aα scaffolding subunit, the PP2A Cα catalytic subunit, and over 20 different regulatory subunits that display differing sensitivities to carbomethylation of the catalytic subunit.** The mechanism of dysregulation of each class of subunits in cancer is illustrated in the figure as well as therapeutic intervention being developed as disease-modifying therapies.

PP2A is widely considered a tumor suppressor and has a fundamental role in regulating the reversible serine/threonine phosphorylation that governs cellular homeostasis (3, 181). It is important to remember that PP2A is in fact a family of more than 60 phosphatases, and the role of each of these heterotrimeric PP2A holoenzymes in cancer is unique, making the picture much more nuanced (Fig. 6). Through cellular and mouse models studying PP2A’s role in cancer, it has been established that distinct PP2A heterotrimers function as tumor suppressors, while others are essential for oncogenic transformation (38, 42, 58, 59, 192, 193, 194). Patient data further support these experimental findings, where PP2A is not found to be completely inactivated in cancer but instead mechanisms exist that bias PP2A heterotrimeric assembly. This bias results in a modulation of PP2A function but not complete inhibition of enzymatic activity. Phosphatases were once considered undruggable given their structural diversity. However, using this diversity as an advantage by targeting PP2A using molecular glues and/or disruptors could allow for the specificity to re-establish PP2A homeostasis for the treatment of a broad range of cancers (20, 44, 47, 64, 194, 195, 196, 197, 198, 199).

Cancer is not a singular disease but instead a series of related diseases that have distinctive disease driving mechanisms. This diversity is reflected in the broad range of mechanisms through which PP2A is dysregulated in cancer (198, 199). A better understanding of the underlying mechanisms by which PP2A is dysregulated in different cancers can be accomplished by integrating different omics approaches together with functional and biological validation of the identified mechanisms of PP2A perturbation in disease. Identifying all PP2A-regulated substrates will be a herculean task, but the information gained will be invaluable to developing heterotrimer-specific approaches to therapeutically modulate its enzymatic function and activity. An integrated approach using structural, biophysical, biochemical, cell-based, and model organism–based research will aid in the development of PP2A-specific drugs for cancer treatment.

## Conflict of interest

G. N. is chief scientific officer at RAPPTA Therapeutics, is an SAB member at Hera BioLabs, reports receiving commercial research support from RAPPTA Therapeutics, and has ownership interest (including patents) in RAPPTA Therapeutics. C. M. O. is a consultant for RAPPTA Therapeutics. The authors declare that they have no conflicts of interest with the contents of this article.
